# Harnessing hyaluronic acid for the treatment of osteoarthritis: A bibliometric analysis

**DOI:** 10.3389/fbioe.2022.961459

**Published:** 2022-09-09

**Authors:** Jun Zhang, Miaoyuan Lin, Yanran Huang, Yuping Wang, Tianji Huang, Zhillin Wu, Zefang Li, Jingtao Xu, Runhan Zhao, Xiaoji Luo

**Affiliations:** ^1^ Department of Orthopedics, The First Affiliated Hospital of Chongqing Medical University, Orthopedic Laboratory of Chongqing Medical University, Chongqing, China; ^2^ Department of Orthopedics, The Affiliated Hospital of Zunyi Medical University, Zunyi, China; ^3^ Department of Orthopedics, Dazhou Central Hospital of Sichuan, Dazhou, China; ^4^ Department of Orthopedics, Qianjiang Central Hospital of Chongqing, Chongqing, China

**Keywords:** Hyaluranic acid, Osteoarthritis, Bibliometrics, HA, OA

## Abstract

**Background:** Osteoarthritis (OA) is a common disease which usually occurs in middle-aged and elderly people. Hyaluronic acid (HA) has been widely used to treat OA and related researches on the efficacy and safety of HA in the treatment of OA have been published. Therefore, the purpose of this research was to investigate the subject characteristics of harnessing HA for the treatment of OA and to analyse the relevant trends and hotspots by using a bibliometric approach.

**Methods:** The articles published from 1 January 2002 to 31 December 2021 were searched in the Web of Science Core Collection (WoSCC) and the relevant information of HA for the treatment of OA was extracted after screening. Then, a total of 2438 publications were analysed by using Microsoft Excel, CiteSpace 5.8.R3, VOSviewer 1.6.18 and the Online Analysis Platform of Literature Metrology (http://bibliometric.com/).

**Results:** A total of 2438 articles were finally included for analysis. The number of publications increased year by year. A total of 83 coutries and 3319 institutions published 2438 manuscripts in the field of use HA for the treatment of OA. The most productive country was United States with total 689 publications and League of European Research Universities Leru (Belgium) was the leading institution with total 126 publicatios. In terms of authors, the most prominent author was KrausVB, who published 28 papers with the highest H-index (19). In addition, Osteoarthritis and Cartilage had the highest number of publications (152 articles) and the highest number of citations (6450 citations). The co-cited references analysis indicated that the article published by McAlindon in 2014 had the most highest number of citations (91co-citations). What’s more, most research hotspots focused on the efficacy and safety of HA, and regenerative medicine researches such as platelet-rich plasma (PRP) and mesenchymal stem cells (MSCs) have attracted more and more attentions of researchers.

**Conclusion:** This study visually analyzed the historical evolution and future trends of HA for the treatment of OA, and discussed the research priorities. At present, there are still different views on the efficacy of HA for the treatment of OA. Gradually, research hotspots of this field have focused on the regenerative medicine.

## Introduction

Osteoarthritis (OA) is a common chronic degenerative disease of the elderly joint, characterized by articular cartilage degeneration, periarticular osteophyte hyperplasia, synovial hyperplasia and subchondral bone changes (Raposo et al., 2021; Zacharjasz et al., 2021). The clinical symptoms of OA include pain, swelling, limited mobility and joint deformity. In severe cases, muscle atrophy, proprioception, and even disability may occur (Raposo et al., 2021; Zacharjasz et al., 2021). It is reported that by 2050, 130 million people will suffer from OA worldwide, of whom 40 million will be severely disabled by the disease (Zacharjasz et al., 2021). The prevalence of OA has been increasing due to an aging population and the increase of the related factors, such as obesity.

Despite the extensive researches on OA, the complex pathophysiology of OA is still not fully understood and there is no golden standard for the treatment of OA. Currently, the “step therapy” has been widely used in the treatment of OA, which includes non-pharmacological treatment, pharmacological treatment and operative treatment. The aim of these treatments is to relieve symptoms of pain and recover the joint function (Cooper et al., 2017). In the early stages of OA, the pharmacological treatment can relieve the symptoms of OA and has been widely used in clinic.

Hyaluronic acid (HA) is a ubiquitous high molecular weight natural glycosaminoglycan, which is composed of repeating units of D-glucuronic acid, N-acetylglucosamine disaccharide linked by B-(1-4) and B-(1-3) glycosides of biopolymers (Salwowska et al., 2016). HA can promote the synthesis of cartilage matrix and improve the elasticity and humidity of cartilage (Moreland and Larry, 2003). It can also inhibit chondrocyte apoptosis and stimulate proteoglycan synthesis (Zhou et al., 2010). Therefore, HA plays an important role in preventing cartilage erosion and reduceing synovial inflammation. In OA patients, the depolymerizations and clearances rate of endogenous HA in joints are higher than normal levels, which not only reduce the concentration and molecular weight of HA, but also reduce the elastic viscosity of synovial fluid (Wang et al., 2004). In United States, HA has been widely used in the daily management of OA. Exogenous supplement of HA can inhibit the degradation of endogenous HA and restore the elasticity and viscosity of synovial fluid (Moreland, 2003; Altman et al., 2015). As a slow delivery drug system, intra-articular injection of HA has became one of the common no-surgical options for the treatment of OA (Tan et al., 2021). For example, Supartz FX is the first clinically approved intra-articular injection of HA product in the word (Altman et al., 2018).

With the wide application of HA in the clinical treatment of OA, the number of related academic papers has also increased dramatically. Over the past two decades, various publications have described the efficacy and safety of HA in the treatment of OA, such as how to prolong the residence time of HA in the joint cavity, the optimal frequency of injection, and the efficacy of different molecular weights of HA. However, a comprehensive and intuitive analysis of the evolution and trend of HA in the treatment of OA by a bibliometric analysis is still lacking. Bibliometric analysis is an important quantitative analysis method for the published papers on a specific topic, which has been widely used in various fields, including mathematics, artificial intelligence, economics and clinical medicine (Chen et al., 2021; Gurnani and Kaur, 2021; Peng et al., 2022). In this study, we used a bibliometric approach to scientifically quantify and quantitatively analyze published articles on HA for the treatment of OA in the WoSCC database from 1 January 2002 to 31 December 2021.

## Materials and methods

### Data collection and retrieval strategy

**FIGURE 1:**
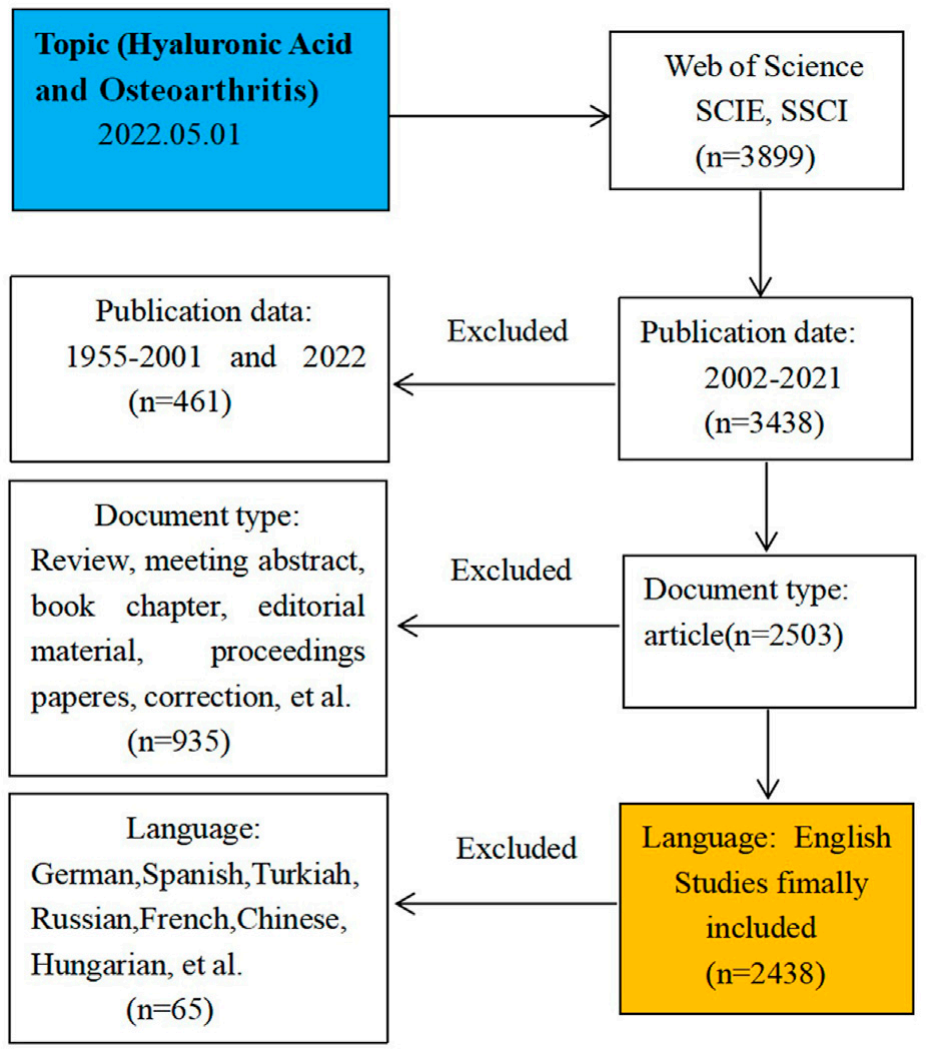
Frame flow diagram of HA for the treatment of OA search strategy from 1 January 2002 to 31 December 2021 based on Web of Science.

This study was conducted on 1 May 2022 and all published data were collected on this day. Although the use of bibliometric approach can scientifically quantify and quantitatively analyze published articles, some constraints associated with bibliometric data should not be ignored in our research, such as the potential bias and incomplete information. These data were downloaded from the Web of Science Core Collection (WOSCC) database and the retrieval strategy was shown in Figure 1. The search formula was TS = (“Hyaluronic Acid” OR “Acid Hyaluronic” OR “Sodium Hyaluronate” OR “Hyaluronate Sodium” OR “Hyaluronate Sodium” OR “Hyaluronan” OR Healon) AND TS = (Osteoarthritis OR Osteoarthrosis OR Osteoarthritides OR Osteoarthroses OR “Arthritis Degenerative” OR “Arthritides Degenerative” OR “Degenerative Arthritides” OR “Degenerative Arthritis”). The document type was “article” and the date of publication was from 1 January 2002 to 31 December 2021. The language was limited to“English”.

### Data analysis and network mapping

Two authors independently screened and extracted all data from the final included articles, including bibliographic information such as publication date, author, country/region, institution, journal, keywords, citation frequency and so on. Visual analysis was performed using Microsoft Excel, CiteSpace 5.8.R3, VOSviewer 1.6.18, the Online Analysis Platform of Literature Metrology (http://bibliometric.com/) after data collection.

### Research ethics

In this study, we searched and downloaded publicly published literature information from WOSCC. This was a data extraction process that did not involve interaction with human subjects or animals. Therefore, the use of these data presented no ethical issues and did not require ethics committee approval.

## Results

### Global annual publications and citations

**FIGURE 2:**
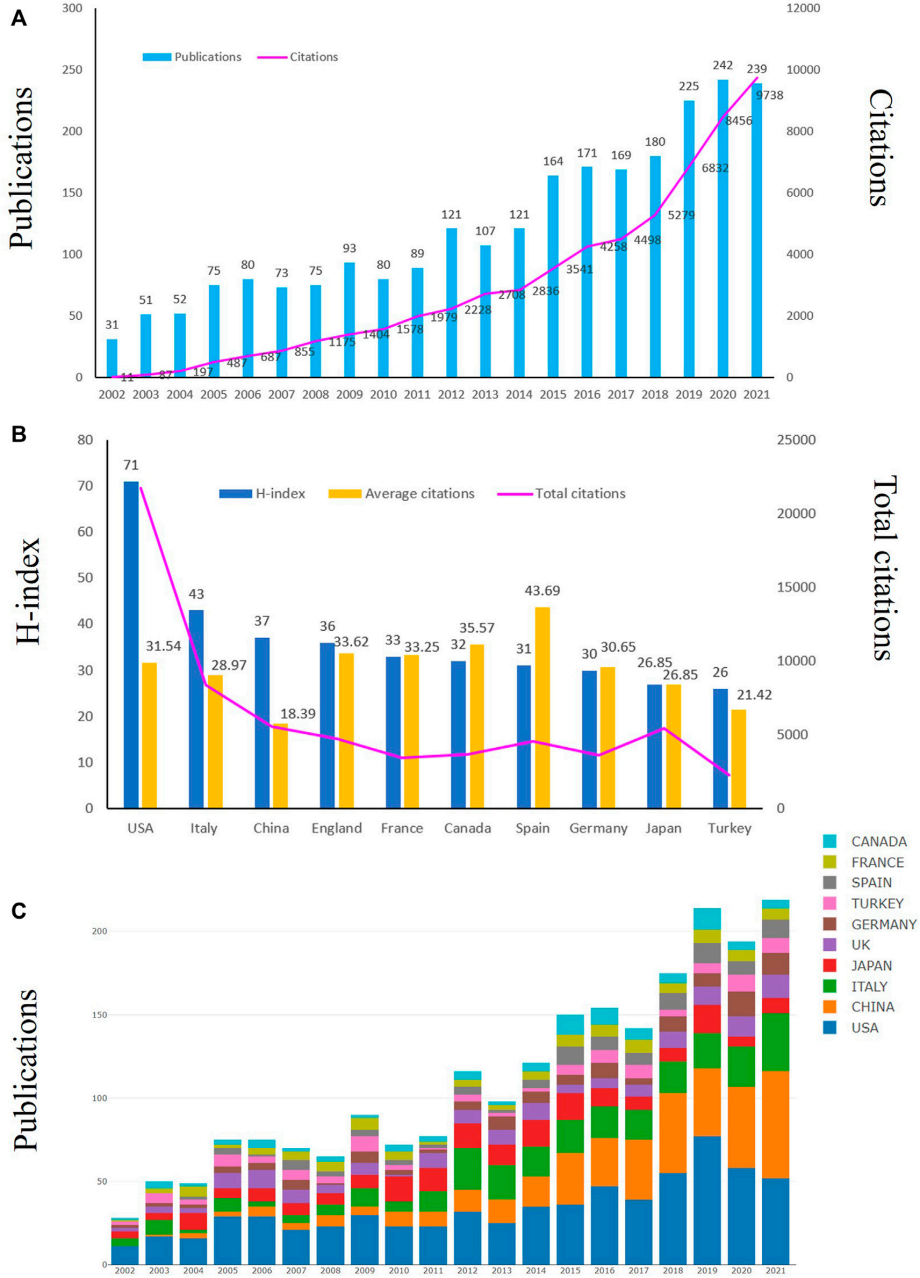
Trends in publications and citations of HA for the treatment of OA. **(A)** The annual trends of global publications and citations. **(B)** The total citations, average citations (citations per article) and H-index of the top ten countries. **(C)** The temporal trends of publications from the top ten countries.

Articles published between 1 January 2002 to 31 December 2021 were retrieved from WOSCC. We initially searched 3899 studies related to HA for the treatment of OA, and finally included 2438 studies according to the inclusion criteria. Figure 2 showed the annual yield growth trend of HA publications for the treatment of OA. According to the annual distribution of publications, interest in HA for the treatment of OA has increased dramatically over the past two decades (Figure 2A). Although some annual publications decreased slightly compared to previous years, the overall trend was still upward. Annual global publications increased from 31 in 2002 to 242 in 2020 and the annual publications could be roughly divided into two stages. In the first phase (2002–2011), annual publications rose slowly and the annual publications were less than 100 per year. In the second stage (2012–2021), there was a rapid increase in the number of published articles, with the annual outputs steadily increasing from 121 to 242. The annual publications peaked in 2020 (242 publications). During the recent 4 years (2018–2021), the total publications of HA for the treatment of OA accounted for 36% (886/2438) of the total published articles. Additionally, the annual number of citations from 2002 to 2021 also increased with the increase of annual publications (Figure 2A). Therefore, the above results indicated that more and more vital researches have been conducted recently and HA for the treatment of OA has got extensive attention from global scholars.

### Distribution of countries/regions

**FIGURE 3:**
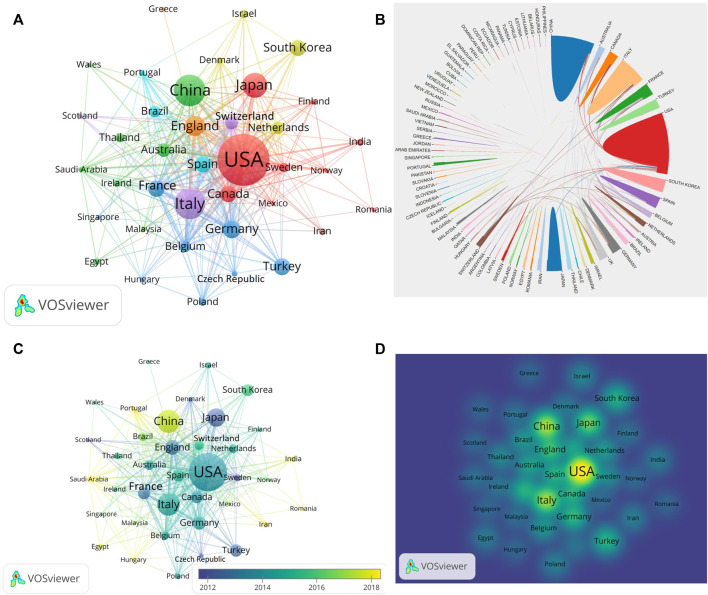
Visualization map of countries involved in HA for the treatment of OA research. **(A)** Collaboration between coutries based on VOSviewer. **(B)** Collaboration between coutries based on Online Analysis Platform of Literature Metrology. **(C)** Dynamics and trends of coutries over time. **(D)** Density map of country distribution of published articles based on VOSviewer.

From 1 January 2002 to 31 December 2021, a total of 2438 publications related to HA for the treatment of OA were published from 83 countries and 3319 institutions. The top ten productive countries were shown in Table 1 and the research outputs of these ten countries accounted for 89% (2158/2438) of the total publications. The most productive country was United States with total 689 publications, followed by China (303 publications), Italy (289 publications), Japan (202 publications) and England (141 publications). In terms of published papers, United States always ranked first in the world. What’s more, United States had the highest total citations (21734) and highest H-index (71), which indicated that United States had the strongest scientific research strength and had an important influence in this field (Figure 2B). China was the second productive country with a total 303 publications and the annual publications surpassed United States in 2021 (Figure 2C). Therefore, we could conclde that United States and China focused on the field of HA for the treatment of OA and made great contribution to this field. The international collaboration network was shown in Figure 3, which demonstrated that the cooperation between global countries was quite close. As shown in Figure 3A, the size of each circle in the graph represented the number of papers published in that country while the lines connecting the circles indicated the degree of cooperation between these countries. We could find United States had close cooperation with China, Japan and Australia (Figures 3A,B). Nevertheless, Greece lacked cooperation with other countries (Figure 3A). Since 2014, the researches in the field of HA for the treatment of OA have increased in United States, Italy and Germany while the researches in this field increased in China, Portugal and Saudi Arabia since 2018 (Figure 3C). The density visualization also demonstrated that United States had the leading role in the field of HA for the treatment of OA (Figure 3D).

**TABLE 1:** The top ten countries that contributed publications on HA for the treatment of osteoarthritis.

Rank	Country	Total publicatios	Total citations	Average citations	H-index
1	United States	689	21734	31.54	71
2	China	303	5573	18.39	37
3	Italy	289	8371	28.97	43
4	Japan	202	5424	26.85	26
5	England	141	4740	33.62	36
6	Germany	118	3617	30.65	30
7	Turkey	106	2271	21.42	26
8	Spain	104	4544	43.69	31
9	Canada	103	3664	35.57	32
10	France	103	3425	33.25	33

### Distribution of institutions

**FIGURE 4:**
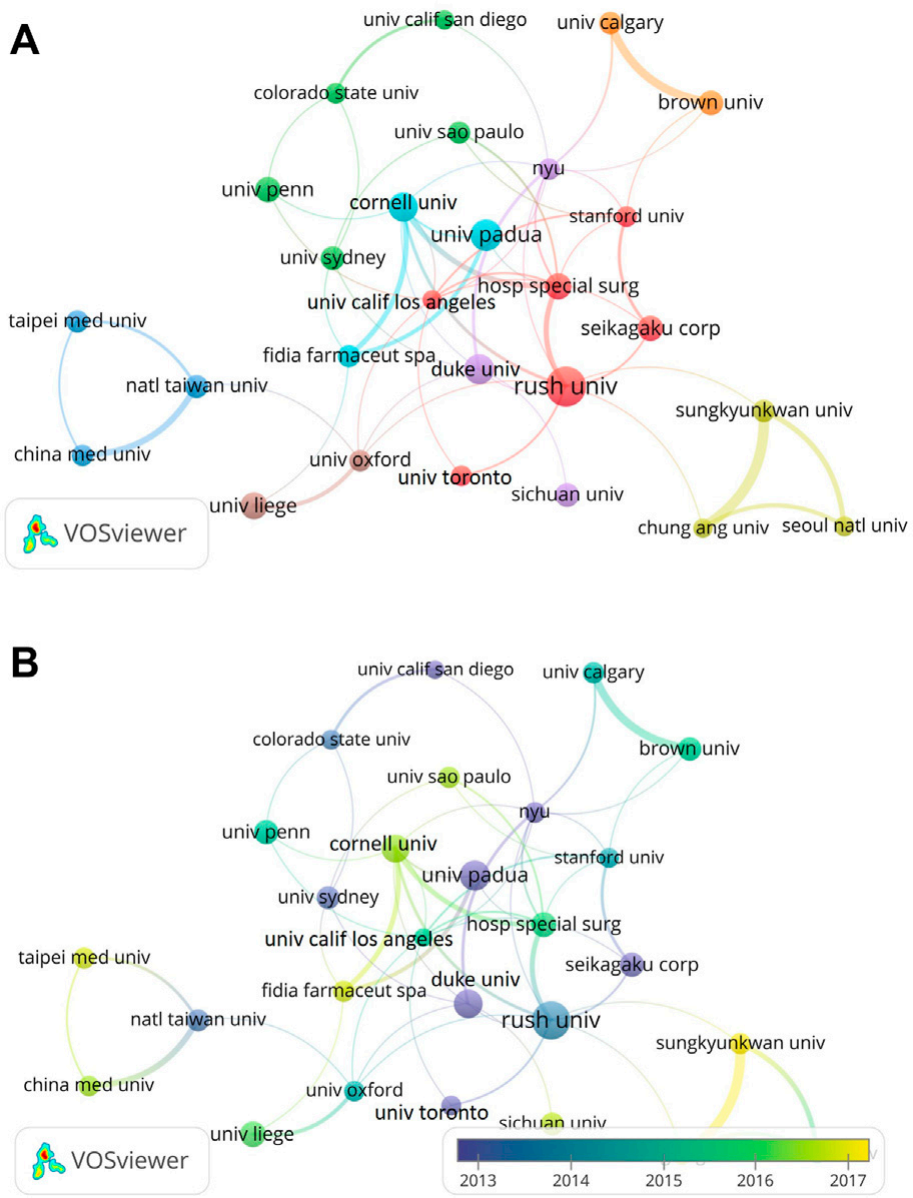
Visualization map of institutions involved in HA for the treatment of OA research. **(A)** Collaboration between institutions involved in HA for the treatment of OA research. **(B)** Dynamics and trends of institutions over time.

A total of 3319 institutions published 2438 articles from 2002 to 2021. Table 2 showed the top ten institutions that published articls on the use of HA for the treatment of OA, including the institution name, country, total number of publications, total citations, average citations and H-index. The most productive institution was League of European Research Universities Leru (Belgium, 126 papers), followed by Rush University (United States, 65 papers), University of California System (United States, 59 papers). Of the top ten institutions, five institutions belonged to United States and three institutions belonged to France. As shown in Figure 4A, we could find all institutions from all over the world cooperated closely with each other. For instance, Rush University cooperated closely with Hospital for Special Surgery, University of California-Los Angeles and Seikagaku Corporation. Some institutions started early researches in this field while other institutions focused on this field in recent years. For example, University Padua, Duke University, Seikagaku Corporation focused on this field since 2013 while the researches in this field increased in Sungkyunkwan University and Taipei Medical University since 2017 (Figure 4B).

**TABLE 2:** The top ten institutions that contributed publications on HA for the treatment of osteoarthritis.

Rank	Institutions	Country	Total publicatios	Total citations	Average citations	H-index
1	League of EuropeanResearch Universities Leru	Belgium	126	4799	37.2	37
2	Rush University	United States	65	2170	33.38	26
3	University of California System	United States	59	2178	36.92	26
4	Assistance Publique Hopitaux Paris Aphp	France	47	1636	34.08	21
5	Udice French Research University	France	42	1545	35.93	20
6	Cornell University	United States	41	1931	47.1	17
7	University of Padua	Italy	38	1297	34.13	19
8	University De Paris	France	36	1004	27.14	16
9	Duke University	United States	34	2219	65.26	22
10	University of North Carolina	United States	32	1,015	31.72	18

### Authors and co-cited authors

**FIGURE 5:**
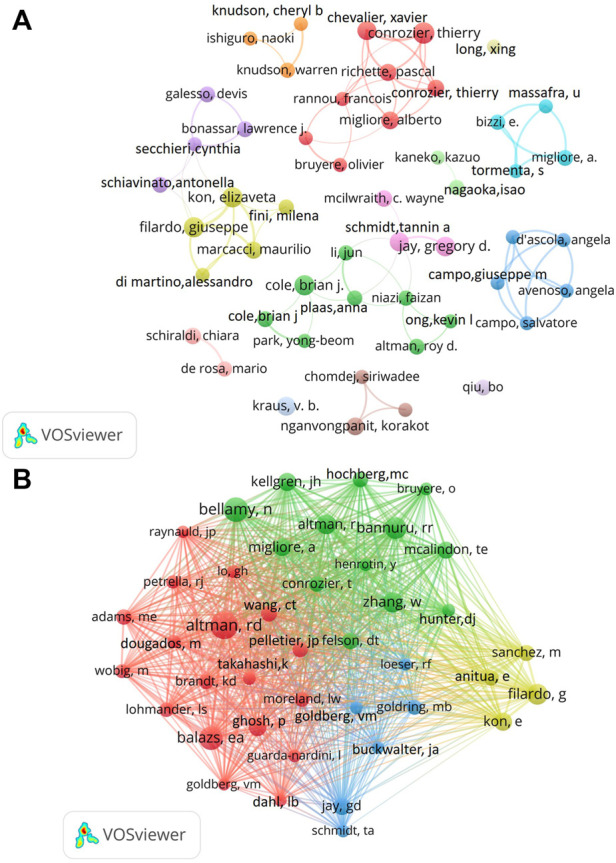
Visualization map of authors and co-cited authors devoted to HA for the treatment of OA research. **(A)** Cooperation network of authors. **(B)** Co-citation network of authors.

From 2002 to 2021, a total of 11920 authors published 2438 articles related to HA for the treatment of OA. Among the top ten productive authors, we could find the most prominent author was KrausVB, who published 28 papers with the highest H-index (19) (Table 3). The second productive author was Conrozier T (26 papers), followed by Migliore A (24 papers). The co-authorship analysis demonstrated that there was a certain network of cooperation among different authors. For instance, Conrozier T cooperated closely with Chevalier X, Richette P and Migliore A (Figure 5A). Co-cited authors are defined as two or more authors who are cited by one or more publications at the same time, and these two or more authors form co-cited relationship. As shown in Figure 5B, among a total of 32016 co-cited authors, Altman RD was the most cited author (cited 686 times), followed by Bellamy N (cited 543 times) and Balazs EA (cited 481 times).

**TABLE 3:** The top ten authors that contributed publications on HA for the treatment of osteoarthritis.

Rank	Country name	Total publicatios	Total citations	Average citations	H-index
1	Kraus VB	28	1738	62.07	19
2	Conrozier T	26	588	22.62	14
3	Migliore A	24	373	15.54	14
4	Kon E	23	1915	83.26	16
5	Filardo G	21	1665	79.29	15
6	Li J	21	451	21.48	12
7	Chevalier X	20	510	25.5	13
8	Schmidt TA	20	901	45.05	13
9	Cole BJ	18	843	46.83	11
10	Jay GD	18	513	28.5	12

### Distribution of journals and co-cited analysis of journals

**FIGURE 6:**
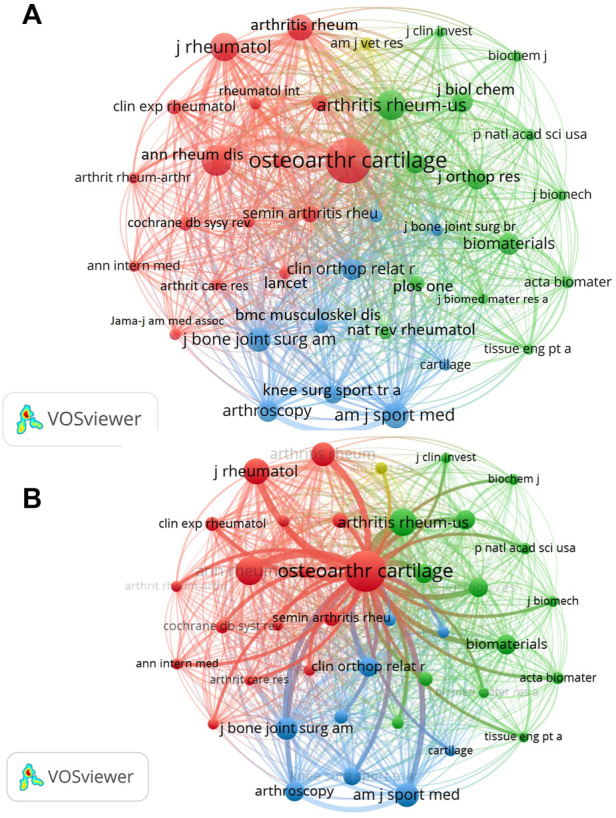
**(A)** Visualization map of co-cited journals of studies related to HA for the treatment of OA. **(B)** The partial enlarged drawing of Osteoarthritis and Cartilage journal.

As for journals, a total of 7604 academic journals published 2438 articles related to HA for the treatment of OA form 2002 to 2021. Table 4 summarized the top ten most active journals that published articles on HA for the treatment of OA. We could find that Osteoarthritis and Cartilage had the highest number of publications (152 articles), followed by Jornal of Orthopedic Research (54 articles), BMC Musculoskeletal Disorders (53 articles), American Journal of Sports Medicine (41 articles) and Arthritis Research Therapy (39 articles). Among the top ten academic journals, Osteoarthritis and Cartilage had the highest impact factor (IF = 6.576). Furthermore, five journals belonged to England while four journals belonged to United States. In addition, four journals belonged to Q1 and three journals belonged to Q2. The impact of journals is also determined by the co-cited times, which reflects whether the journal has a significant impact in a particular field of research. Therefore, we performed co-cited analysis of total 7604 journals by VOSviewer software (Figure 6). The journals with the highest number of citations was Osteoarthritis and Cartilage (6450 citations), followed by Annals of the Rheumatic Diseases (2943 citations) and Arthritis Rheumatology (2794 citations).

**TABLE 4:** Top ten most active journals that published articles on HA for the treatment of osteoarthritis.

Rank	Journal	Articles counts	Country	JCR	IF	Total citations	Average Number of citations	H-index
1	Osteoarthritis and Cartilage	152	England	Q1	6.576	7502	49.36	48
2	Jornal of Orthopaedic Research	54	England	Q1	3.494	1058	19.59	20
3	BMC Musculoskeletal Disorders	53	England	Q2	2.362	1087	20.51	17
4	American Journal of Sports Medicine	41	United States	Q1	6.202	2857	69.68	25
5	Arthritis Research Therapy	39	England	Q2	5.156	1511	38.74	23
6	Cartilage	39	United States	Q4	4.634	502	12.87	12
7	Plos One	39	United States	Q2	3.240	953	24.44	19
8	Clinical Rheumatology	35	England	Q3	2.980	665	19	16
9	Knee Surgery Sports Traumatology Arthroscopy	34	Germany	Q1	4.342	1,400	41.18	20
10	Arthritis And Rheumatism	30	United States	-	-	2,719	90.63	24

**FIGURE 7:**
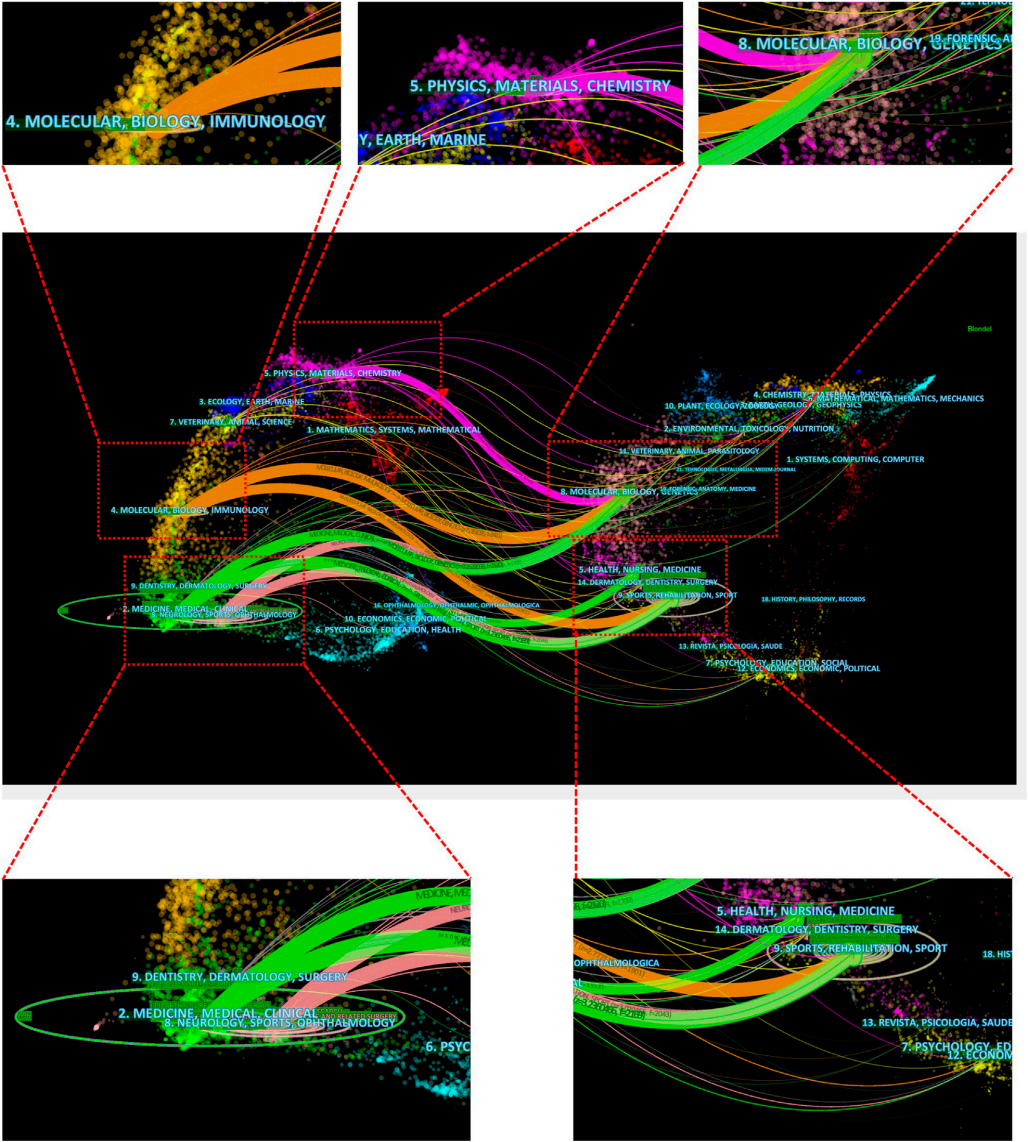
The dual-map overlay of journals related to HA for the treatment of OA based on CiteSpace.

In addition, the dual-map overlay of journals was constructed by Citespace software to evaluate the relationship distribution among journals. As shown in Figure 7, the citing journals were on the left and the cited journals were on the right, and the colored paths between them indicated the citation relationship. There were eight paths in Figure 7, including one purple path, two orange paths, three green paths, and two pink paths. The purple path indicated that papers published in Physics, Materials and Chemistry typically cited papers published in Molecular, Biology and Genetics. The upper orange path presented that papers published in Molecular, Biology and Immunology always cited papers published in Molecular, Biology and Genetics. The lower orange path demonstrated that articles published in Molecular, Biology and Immunology often cited articles published in Sports, Rehabilitation and Sport. The upper green path demonstrated that articles published in Medicine, Medical and Clinical often cited articles published in Molecular, Biology and Genetics. The lower green path demonstrated that articles published in Medicine, Medical and Clinical often cited articles published in Sports, Rehabilitation and Sport. The pink paths showed that articles published in Neurology, Sports and Ophthalmology frequently cited articles published in Molecular, Biology, Genetics, Sports, Rehabilitation and Sport.

### Co-cited references analysis

**FIGURE 8:**
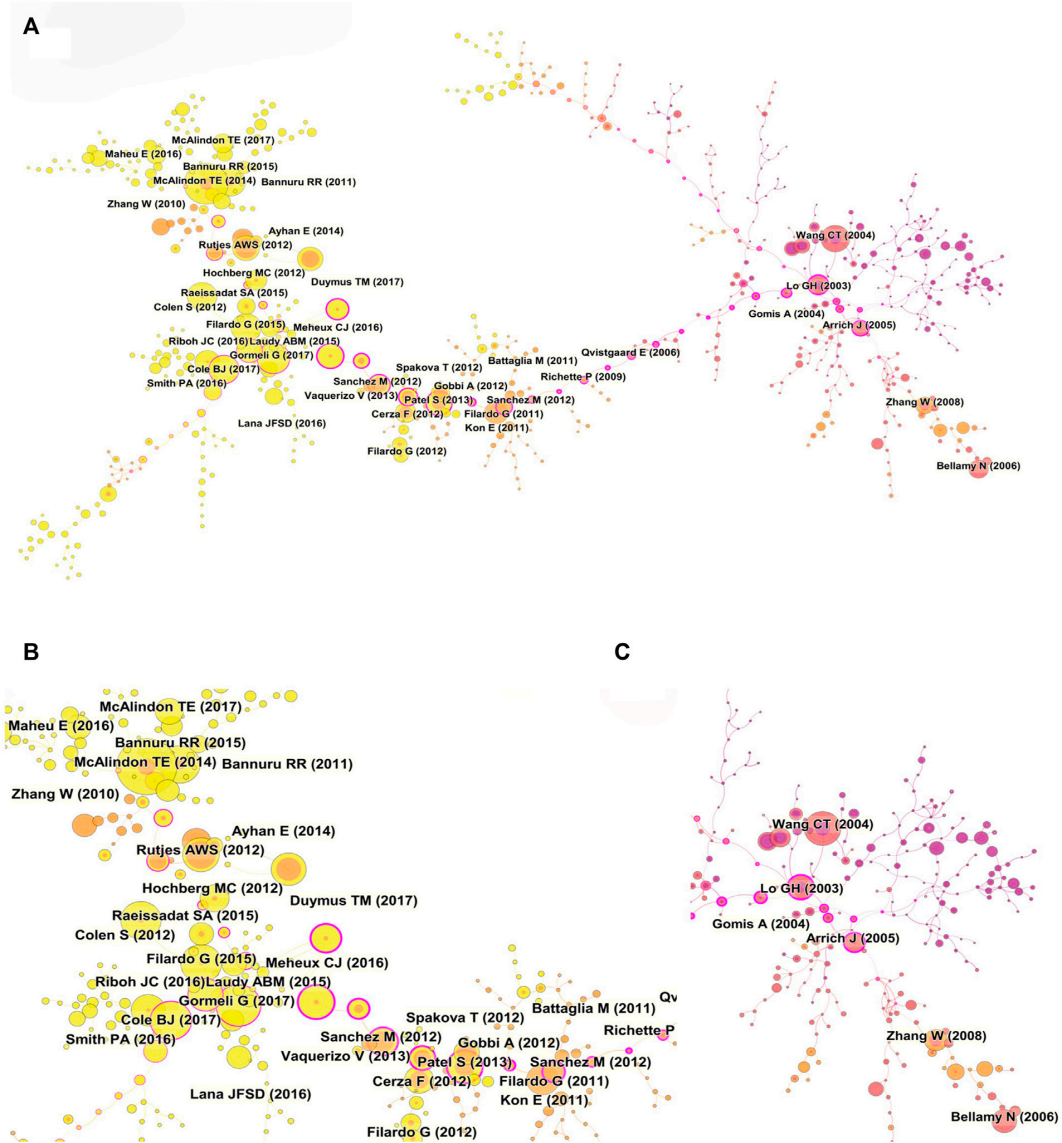
**(A)** Visualization map of co-cited references analysis based on CiteSpace. **(B**, **C)** The partial enlarged drawings of panel A.

**FIGURE 9:**
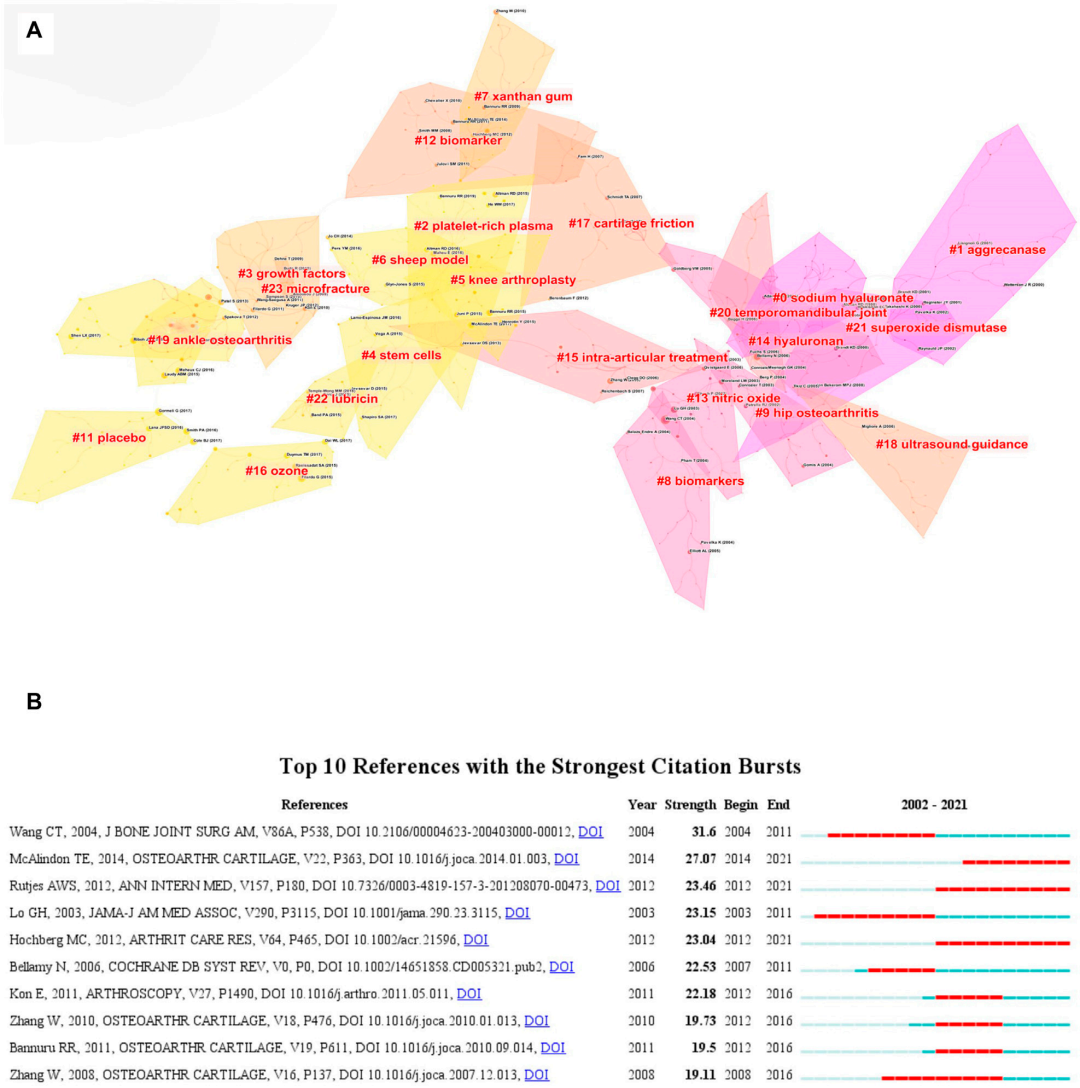
**(A)** Cluster analysis of co-cited references based on CiteSpace. **(B)** Visualization map of top ten references with the strongest citation bursts related to HA for the treatment of OA based on CiteSpace.

Reference co-citation refers to an article is cited by one or more papers at the same time and the two papers are considered to be a co-citation relationship. Reference co-citation is a research method to measure the degree of relationship between the articles. The paper with high co-citation indicates it has a significant influence in a certain field (Wu et al., 2021a). Table 5 listed the ten most frequently cited references. Among the top ten most cited references, two references belonged to guidelines, four references belonged to randomized clinical trials, and four references belonged to systematic reviews. Each of the top ten co-cited references was co-cited at least 53 times. It was worth noting that most of the early references focused on the efficacy of HA treatment, and four recently published clinical trials reported the therapeutic efficacy of platelet-rich plasma (PRP) (Raeissadat et al., 2015; Cole et al., 2017; Görmeli et al., 2017; Di Martino et al., 2019). More and more researches compared the therapeutic effect of HA and PRP, although the final conclusion was still undefined. Among the references with high citations, the paper published by McAlindon TE in 2014 had the most highest number of citations (91 citations) (Bannuru et al., 2019). The reference with the second high number of citations was published by Gormeli G (67 citations) (Görmeli et al., 2017), followed by the article that was published by Bannuru RR (63 citations) (Bannuru et al., 2015), Filardo G (63 citations) (Filardo et al., 2015), Cole BJ (62 citations) (Cole et al., 2017). We also constructed a co-citation visualization network of references by using Citespace software. As shown in Figure 8, the size of the circles represented the co-citation times of a paper. The circle with a purple outer ring indicated this paper had high centrality and significant influence on a certain field. Then, the cluster map of the co-cited references was constructed with Citespace software. As shown in Figure 9A, a total of 22 clusters were formed and the top ten cluster was “#0 sodium hyaluronate”, followed by “#1 aggrecanase”, “#2 platelet-rich plasma”, “#3 growth factors”, “#4 stem cells”, “#5 knee arthroplasty”, “#6 sheep model”, “#7 xanthan gum”, “#8 biomarkers”, “#9 hip osteoarthritis”.

**TABLE 5:** The top 10 co-cited references related to HA for the treatment of osteoarthritis.

Rank	Author	Citations	Title	Journal	Year
1	McAlindon TE	91	OARSI guidelines for the non-surgical management of knee osteoarthritis	Osteoarthritis Cartilage	2014
2	Gormeli G	67	Multiple PRP injections are more effective than single injections and hyaluronic acid in knees with early osteoarthritis: a randomized, double-blind, placebo-controlled trial	Knee Surg Sports Traumatol Arthrosc	2017
3	Bannuru RR	63	Comparative effectiveness of pharmacologic interventions for knee osteoarthritis: a systematic review and network meta-analysis	Ann Intern Med	2015
4	Filardo G	63	Platelet-Rich Plasma Intra-articular Knee Injections Show No Superiority Versus Viscosupplementation: A Randomized Controlled Trial	Am J Sports Med	2015
5	Cole BJ	62	Hyaluronic Acid Versus Platelet-Rich Plasma: A Prospective, Double-Blind Randomized Controlled Trial Comparing Clinical Outcomes and Effects on Intra-articular Biology for the Treatment of Knee Osteoarthritis	Am J Sports Med	2017
6	Raeissadat SA	61	Knee Osteoarthritis Injection Choices: Platelet- Rich Plasma (PRP) Versus Hyaluronic Acid (A one-year randomized clinical trial)	Clin Med Insights ArthritisMusculoskelet Disord	2015
7	Wang CT	60	Therapeutic effects of hyaluronic acid on osteoarthritis of the knee. A meta-analysis of randomized controlled trials	J Bone Joint Surg Am	2004
8	Rutjes AWS	56	Viscosupplementation for osteoarthritis of the knee: a systematic review and meta-analysis	Ann Intern Med	2012
9	Altman RD	55	The mechanism of action for hyaluronic acid treatment in the osteoarthritic knee: a systematic review	BMC Musculoskelet Disord	2015
10	Hochberg MC	53	American College of Rheumatology 2012 recommendations for the use of nonpharmacologic and pharmacologic therapies in osteoarthritis of the hand, hip, and knee	Arthritis Care Res	2012

In addition, the top ten references with the strongest strength citation burst were constructed by CiteSpace. As shown in Figure 9B, the length of the lines in the graph represented the time period from 2002 to 2021 while the red line represented the time interval of citation bursts. The burst strength of the top ten publications related to HA for the treatment of OA ranged from 19.11 to 31.6 and the endurance strength ranged from 5 to 10 years. Among the top ten references, the paper with the highest citation burst was published by Wang CT in 2004 (Wang et al., 2004). In this paper, Wang CT et al. used a mate-analysis to describe the relevant efficacy of HA in the treatment of KOA. The results confirmed that intra-articular injection of HA could reduce the symptoms of KOA. The patients’ pain and functional outcomes were significantly improved. The paper with the second citation burst was a guideline on the treatment of KOA, which was published by McAlindon TE in 2014. This paper put forward 29 treatments for different clinical subtypes of KOA (McAlindon et al., 2014). Surprisingly, a high-quality mate-analysis published by Rutjes AWS indicated that HA had no clinical benefit in the treatment of OA and increased the risk of serious adverse events (Rutjes et al., 2012).

### Co-occurrence analysis of keywords

**FIGURE 10:**
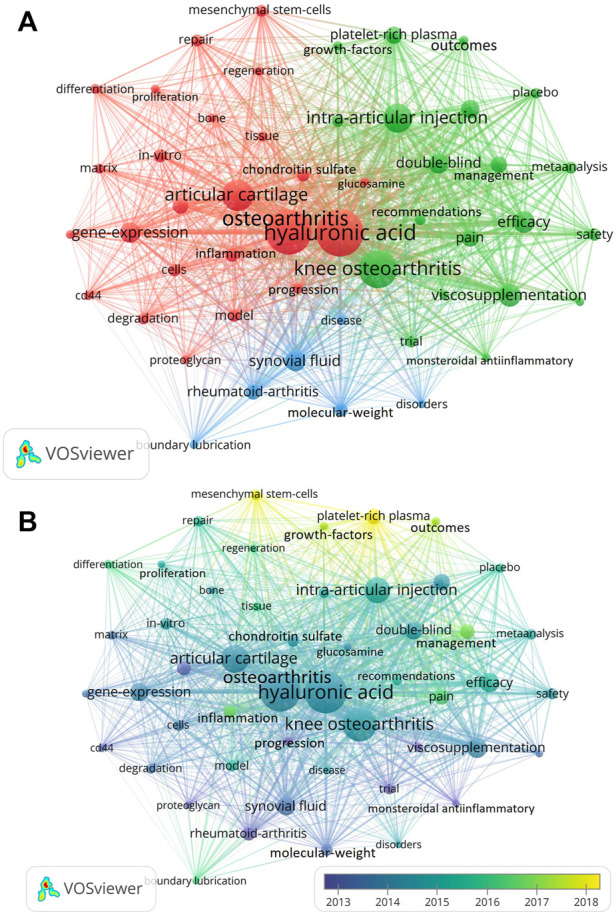
Mapping of keywords in studies on HA for the treatment of OA based on VOSviewer. **(A)** Network visualization of keywords. **(B)** Chronological order of keywords.

The analysis of hot words and frontier words is the core of the published paper and we can summary the research focuses and find the hot topics in a certain field. Keywords as one of the important parts of a article present the main topic of the papers. Therefore, we analyzed the keywords of the published paper and conducted the map networks by using Vosviewer software. A total of 6599 keywords were extracted and analysed in this study. As shown in Figure 10A, a total of 50 keywords appeared in the picture (defined as keywords that were utilized more than 57 times in titles or abstracts in all publications). The keywords with high occurrence frequency were “hyaluronic acid” (1692), “osteoarthritis” (1544), “knee osteoarthritis” (1079), “articular cartilage” (749), “intra-articular injection” (620), “viscosupplementation” (371), “efficacy” (348), “gene-expression” (325), “synovial fluid” (315) and “double-blind” (302). These keywords were mainly divided into 3 clusters and the different color represented different category. The red cluster (including 24 keywords) mainly represented biological research, and the main keywords were “hyaluronic acid”, “osteoarthritis”, “articular cartilage” and “gene-expression”. The green cluster (including 20 keywords) primarily represented clinical research, and the main keywords included “knee osteoarthritis”, “intra-articular injections”, “double-blind”, “hip osteoarthritis”, “viscose supplementation”, “efficacy” and so on. The blue cluster (including 6 keywords) typically indicated the other researches related to HA for the treatment of OA, which included “synovial fluid”, “rheumatoid-arthritis”, “molecular-weight”, “disease”, “boundary lubrication” and “disorders”. Then, the colors of the keywords were classified by VOSviewer according to the average publication year (APY). As shown in Figure 10B, the keywords in yellow suggested they appeared later than those keywords in blue. The results indicated that “PRP”, “mesenchymal stem cells (MSCs)" and “growth factor” were the recent primary topics in the field of HA for the treatment of OA.

## Discussion

In this study, we used bibliometric analysis to systematically evaluate the development and trend in the field of HA for the treatment of OA, which could help us to discover milestone achievements and find new research hotspots. For instance, HA and PRP for the treatment of OA have always been the research focus of researchers, although the therapeutic effect and final conclusion are still undefined. Therefore, this will be a crucial research direction in the future researches not only in basic researches but also in clinical research. In addition, MSCs treatment for OA has made some important achievements, which has been used in clinic to treat OA. The number of papers published in this field increased during the past two decades. Since 2012, the speed of publications has increased rapidly and entered a period of rapid development.

United States was the most productive country with the highest citations, and the total citations of United States were more than twice of the other countries, which indicated that United States was at the forefront of scientific research and made great contribution in the field of HA for the treatment of OA. Although China ranked second in terms of the total publications, the average citation was low (18.39). Therefore, more high-quality articles are needed in future researches. The influence and contribution of scientific research institutions represented the scientific research level of a country or region. Of the top ten productive institutions, five institutions were in United States, three institutions were in France, and the remaining three institutions were from Belgium and Italy. It was worth noting that League of European Research Universities Leru (Belgium) was the most productive institution, which published 126 papers related to HA for the treatment of OA. This institute focused on the management of non-drug, drug and surgical treatment of OA. Their recent research demonstrated that HA not only could be applied to KOA and hip osteoarthritis (HOA), but also could be used to thumb-base osteoarthritis (TBOA) (Tenti et al., 2021).

Different journals include different fields of publication. We can find the most productive journals and most influential journals in a certain field by the bibliometric approach, which is capable of providing the value of guidance for researcheds in this field. Among the top ten most productive journals, six journals belonged to the field of orthopedics, two journals belonged to the field of rheumatology, one journal belonged to the field of biology, and one journal belonged to the field of sports medicine. This indicated that OA was the focus of multidisciplinary researches. Osteoarthritis and Cartilage (IF6.576) had the highest number of published articles and citations, indicating that this journal was the most popular journal for scholars focusing on OA. Scholars from all over the world hope to publish landmark articles in this journal, because this journal represents the most advanced achievements and major breakthroughs in OA research. In recent years, this journal has been mainly devoting to exploring the breakthrough of the basic researches and treatment methods of OA. Recently, Felson et al. found that the level of fatty acids in blood was not associated with the risk of late KOA, which broke through the previous belief that obesity increased the incidence of OA (Felson et al., 2021).

In terms of authors who had contributed to HA for the treatment of OA, we found that Kraus VB was the most productive scholar with a total of 28 publications. His team has been committed to a series of studies on the pathogenesis, biomarkers, treatment and rehabilitation of OA. In recent years, his team found that biological markers such as VCAM-1, MMP-3, SVCAM-1, sicAM-1, TIMP-1 and VEGF in the articular cavity of OA patients were related to synovial inflammation and severity of symptoms (Haraden et al., 2019). In addition, they used single-cell RNA sequencing to elucidate molecular crosstalk between cartilage and synovial membrane in OA (Chou et al., 2020). It was worth noting that although Kon E was not the most productive author, he ranked first in terms of total citations and average citations (83.26), suggesting that his published papers had significant influence in this field. His team not only focused on HA for the treatment of OA, but also proposed that PRP could alleviate OA by reducing inflammation and promoting tissue anabolism (Kon et al., 2020). Altman RD also carried out a lot of researches in the field of OA. His researches involved not only OA, but also various rheumatic diseases. What’s more, His team has conducted many explorations on the efficacy and safety of HA (Li et al., 2015; Kolasinski et al., 2019).

In addition, among the top ten highly cited literatures, the “OARSI Guidelines for Non-surgical Treatment of Knee Osteoarthritis” had the highest citations (91), which indicated that timely and effective update of guidelines could contribute to the development of this field. Besides, guidelines of a field could providie the best evidence-based information to assist clinician to make treatment decisions. A systematic review focused on the efficacy of HA therapy and demonstrated its safety in symptomatic patients with KOA (Miller et al., 2021). Intraarticular injection HA could alleviate pain and improve knee function, which received explicit recommendations from national and international professional associations (Wang and Yu, 2019). For instance, HA has been recommended by the International Society for the Study of Osteoarthritis (OARSI) (Zhang et al., 2008), the European League against Rheumatism (EULAR) (Jordan et al., 2003) and the American College of Rheumatology as a first-line treatment for OA (Recommendations for the medical management, 2000).

The co-occurrence analysis of keywords demonstrated that keywords with high occurrence frequency was “hyaluronic acid” (1692), “osteoarthritis” (1544), “knee osteoarthritis” (1079), “articular cartilage” (749), “intra-articular injection” (620), “viscosupplementation” (371), “efficacy” (348), “gene-expression” (325), “synovial fluid” (315) and “double-blind” (302). In terms of the efficacy of HA, it was important to pay attention to the selection of different products. Different HA products had different characteristics, including the source of HA (animal and biological fermentation), molecular weight, molecular structure (non-crosslinking, crosslinking or a mixture), crosslinking method, concentration, injection volume. Wu et al. suggested that higher concentration of HA might provide better effects of cartilage protection, proteoglycan/glycosaminoglycan synthesis, anti-inflammatory and analgesia (Wu et al., 2021b). Ong et al. demonstrated that there was no clear correlation between poultry-derived or cross-linked HA and severe acute local reaction (SALR), such as Hylan G-F-20 and non-Hylan G-F-20 (Ong et al., 2021). However, Chen et al. reported adverse reactions of high-molecular weight cross-linked HA in poultry, such as pseudosepsis and severe acute inflammatory reactions (Chen et al., 2002).

What’s more, we could also find that “PRP”, “MSCs” and “growth factor” were research hotspots in recent years. PRP is the plasma component of the whole blood that contains a high concentration of various growth factors. Gokay et al. pointed out that a single dose of PRP or HA had the same efficacy while multiple injections of PRP were conducive to better clinical effect for early OA patients (Görmeli et al., 2017). Both PRP and HA could alleviate OA symptoms by reducing synovial hyperplasia and regulating cytokine levels. What’s more, PRP could also be used as cell growth promoter and cartilage differentiation agent, which could improve the cartilage tissue structure to a certain extent (Mix et al., 2004). In terms of MSCs, various MSCs were used to treat OA owing to their multilineage differentiation potential (especially into chondrocytes), their capacity for selfrenewal and their immunomodulatory properties (Harrell et al., 2019). However, the mechanisms of the effect of MSCs were still largely undefinite, in part because only low-quality evidence was available from preclinical studies (Dan et al., 2018). Currently, most clinical trials of MSCs for the treatment of OA only focused on KOA. Although some results showed the improvement of pain or function in patients treated with MSCs, no convincing evidence of effect on cartilage thicknessas measured by MRI (Pullig et al., 2016; Karimi et al., 2018; Kim et al., 2019; Lee et al., 2019b; Shah et al., 2019).

HA is a glycosaminoglycan that serves as a primary component for proteoglycans of the extra-cellular matrix (ECM). HA is capable of preserving the integrity of cartilage surfaces by providing shock absorbance as well as joint lubrication. Although no evidence has indicated HA can effectively reverse OA progression, HA can stimulate endogenous HA synthesis and release endogenous antinociceptive and antiinflammatory propertie, which can effectively attenuates symptom of OA patients. HA has been conditionally recommended for the treatment of knee OA by The Osteoarthritis Research Society International (OARSI) group (Bannuru et al., 2019). During the past two decades, many researches not only focused on the use of HA for the treatment of OA, but also focused on compare the therapeutic effect of HA and other agents, including PRP, corticosteroid and so on.

Campbell et al. demonstrated that HA was a viable option for knee OA compared with non-steroidal anti-inflammatory drugs (NSAIDs), corticosteroid injections (CSI), PRP, and placebo. The improvements in knee pain and function could persist for up to 26 weeks when HA was used. They indicated that HA had a good safety profile, and use of HA should be considered in patients with early knee OA (Campbell et al., 2015). A systematic review and network meta-analysis performed by Zhao et al. found that HA had inferior therapeutic effect on pain relief and functional improvement when compared with PRP, adipose mesenchymal stem cells and bone marrow mesenchymal stem cells (Han et al., 2021).

PRP is another widely used therapeutic agent for the treatment of OA, which is defined as an autologous formulation from whole blood that is centrifuged to extract a solution with a high platelet concentration. A randomized, double-blind, triple-parallel, placebo-controlled clinical trial demonstrated that intra-articular injection of PRP was superior to HA or saline in the treatment of mild to moderate knee OA (Lin et al., 2019). Kon et al. carried out a prospective comparative level II study that compared the effect of PRP with high-molecular-weight and low-molecular-weight HA, and the results showed that autologous PRP injection demonstrated longer and more efficacy than HA injection in reducing symptoms and pain and recovering articular function. In addition, better results were achieved in younger and more active patients with a low degree of cartilage degeneration, whereas a worse outcome was obtained in more degenerated joints and in older patients, in whom results similar to those of viscosupplementation have been observed (Kon et al., 2011). In another randomized double blind controlled trial, Louis et al. reported that the single injection of pure PRP could obtain a significant clinical improvement in the management of knee OA, which was equivalent to a single HA injection in this patient population (Louis et al., 2018).

Recently, HA-PRP conjugate has been a new and promising therapy for the treatment of OA, which combines the elastic and lubricated properties of HA and the chondroprotective and reparative biologic effects of PRP (Chen et al., 2014). Lee et al. demonstrated that the use of HA-PRP conjugate for the treatment of OA might be superior, which provided significantly better long-term cartilage preservation than HA (Lee et al., 2019a). Chen et al. found that HA + PRP conjugate could retrieve pro-inflammatory cytokines-reduced articular chondrocyte proliferation and chondrogenic phenotype, the mechanism of which involved the sequential activation of specific receptors CD44 and TGF-βRII, downstream mediators Smad2/3 and Erk1/2, and the chondrogenic transcription factor SOX9 (Chen et al., 2014). In addition, the use of HA + PRP conjugate could enhance chondrocyte viability and proliferation while decrease apoptosis, which mainly attributed to the ability of HA-PRP to augment anti-inflammatory and antioxidative chondrocyte proliferation, along with inhibition of MMP-1 activity and matrix calcification (Chiou et al., 2018).

During the past two decades, various biomaterials based on HA have been applied to OA, including hydrogel, hydrogel microsphere, and so on. Among these biomaterials, HAMA was widely used for the treatment of OA, which was synthesized by using HA and methacrylate anhydride (MA) with chemical bond. Huang et al. prepared highly monodisperse photo-crosslinked HAMA microspheres by microfluidic method and transforming growth factor-beta3 (TGF-β3) and platelet-derived growth factor-BB (PDGF-BB) were non-covalently incorporated within the HAMA microspheres by binding heparin. The HAMA microspheres containing TGF-β3 and PDGF-BB could not only recruit endogenous stem cells but also promote chondrogenic differentiation, which had significant therapeutic effect on OA (Lei et al., 2021a). In another study, rapamycin-loaded hydrogenated soy phosphatidylcholine liposomes were integrated into a HAMA matrix *via* non-covalent interactions, forming injectable cationic liposome-incorporating HAMA microspheres, which had cellular homeostasis maintenance and self-renewing hydration lubrication (Lei et al., 2021b). Cui et al. constructed a injectable adhesive HAMA microspheres loading charge-guided nanosized secondary structure (PDA@Lipo@HAMA microspheres). In a rat OA model, the PDA@Lipo@HAMA microspheres could effectively penetrate cartilage matrix and delivered the therapeutic drug to chondrocytes in the oxidative stress environment, which could alleviate OA progression by inhibiting the apoptosis of chondrocytes (Lin et al., 2021).

This study also had some limitations. Firstly, the data extracted and analyzed in this study were from noly WoSCC database although WoSCC is the most commonly used database with high reliability for scientometric studies. Secondly, the potential bias might occur because all data were extracted with software tools. Lastly, the publications published in 2022 were not included in our study, because these data were incomplete at the time of our database search.

## Conclusion

To our knowledge, this study was the first comprehensive analysis of HA for the treatment of OA by using a bibliometric approach. In summary, this study fully summarized and systematically analysed the global research trends related to HA for the treatment of AO from 1 January 2002 to 31 December 2021. The annual outputs of related papers has grown remarkably, and the current research hotspots are the safety and effect of HA for the treatments of OA. Gradually, research hotspots of this field have focused on the regenerative medicine.

## Data Availability

The original contributions presented in the study are included in the article/supplementary material, further inquiries can be directed to the corresponding author.
